# Uptake of prenatal diagnostic testing for retinoblastoma compared to other hereditary cancer syndromes in the Netherlands

**DOI:** 10.1007/s10689-016-9943-z

**Published:** 2016-11-08

**Authors:** Charlotte J. Dommering, Lidewij Henneman, Annemarie H. van der Hout, Marianne A. Jonker, Carli M. J. Tops, Ans M. W. van den Ouweland, Rob B. van der Luijt, Arjen R. Mensenkamp, Frans B. L. Hogervorst, Egbert J. W. Redeker, Christine E. M. de Die-Smulders, Annette C. Moll, Hanne Meijers-Heijboer

**Affiliations:** 1 Department of Clinical Genetics, VU University Medical Center, PO Box 7057, 1007 MB Amsterdam, The Netherlands; 2 Department of Genetics, University Medical Center Groningen, University of Groningen, Groningen, The Netherlands; 3 Department of Mathematics, Faculty of Sciences, VU University, Amsterdam, The Netherlands; 4 Department for Health Evidence, Radboud University Medical Center, Nijmegen, The Netherlands; 5 Department of Clinical Genetics, Leiden University Medical Center, Leiden, The Netherlands; 6 Department Clinical Genetics, Erasmus Medical Center, Rotterdam, The Netherlands; 7 Department of Medical Genetics, University Medical Center Utrecht, Utrecht, The Netherlands; 8 Department of Human Genetics, Radboud University Medical Center, Nijmegen, The Netherlands; 9 Department of Pathology, The Netherlands Cancer Institute, Amsterdam, The Netherlands; 10 Department of Clinical Genetics, Academic Medical Center, University of Amsterdam, Amsterdam, The Netherlands; 11 Department of Clinical Genetics, Maastricht University Medical Centre, Maastricht, The Netherlands; 12 Department of Ophthalmology, VU University Medical Center, Amsterdam, The Netherlands

**Keywords:** Retinoblastoma, Prenatal diagnosis, Von Hippel–Lindau disease, Li–Fraumeni syndrome, Familial adenomatous polyposis, Hereditary breast ovarian cancer

## Abstract

Since the 1980s the genetic cause of many hereditary tumor syndromes has been elucidated. As a consequence, carriers of a deleterious mutation in these genes may opt for prenatal diagnoses (PND). We studied the uptake of prenatal diagnosis for five hereditary cancer syndromes in the Netherlands. Uptake for retinoblastoma (Rb) was compared with uptake for Von Hippel–Lindau disease (VHL), Li–Fraumeni syndrome (LFS), familial adenomatous polyposis (FAP), and hereditary breast ovarian cancer (HBOC). A questionnaire was completed by all nine DNA-diagnostic laboratories assessing the number of independent mutation-positive families identified from the start of diagnostic testing until May 2013, and the number of PNDs performed for these syndromes within these families. Of 187 families with a known Rb-gene mutation, 22 had performed PND (11.8%), this was significantly higher than uptake for FAP (1.6%) and HBOC (<0.2%). For VHL (6.5%) and LFS (4.9%) the difference was not statistically significant. PND for Rb started 3 years after introduction of diagnostic DNA testing and remained stable over the years. For the other cancer syndromes PND started 10–15 years after the introduction and uptake for PND showed an increase after 2009. We conclude that uptake of PND for Rb was significantly higher than for FAP and HBOC, but not different from VHL and LFS. Early onset, high penetrance, lack of preventive surgery and perceived burden of disease may explain these differences.

## Introduction

Approximately 5% of all cancers are caused by a genetic predisposition, with the mode of inheritance being mainly autosomal dominant. In the past 30 years the genetic cause of many hereditary cancer syndromes has been unravelled. Knowledge of the genetic predisposition can aid early diagnosis and management of cancer for affected mutation carriers. For unaffected family members, presymptomatic DNA diagnosis of the disease-associated mutation may enable informed choices about cancer screening or risk reduction strategies, including preventive surgery. Cancer genetic testing, however, can also affect reproductive decisions of mutation carriers [1–5]. Reproductive options for couples at risk of having a child with a cancer risk predisposition may be: refraining from having children or accepting the risk, adoption or gamete donation. Other options may include preimplantation genetic diagnosis (PGD) (i.e. in vitro fertilization, with genetic testing of 1 or 2 cells of the embryo and transfer of unaffected embryos to the uterus) and prenatal diagnostic testing for the deleterious cancer gene mutation with the option to terminate the pregnancy in the case of a carrier foetus. Prenatal diagnosis (PND) and PGD for hereditary cancer syndromes were described as early as 1988 and 1998, respectively [6, 7]. Legal aspects of these two techniques, and thus availability, differ across countries; e.g. in some countries PND followed by abortion is not allowed, whereas in other countries PGD is prohibited [8, 9]. Access to PND and/or PGD is also limited in some countries because they are not covered by health insurances [9]. Both in society and in medical literature, PND and PGD for hereditary cancer syndromes have led to ethical, social and legal discussions [10–15]. Issues under debate are that many cancer-predisposing mutations have incomplete penetrance and that the onset of disease often does not occur until early adulthood. Furthermore, some argue that through the early detection of cancer or preventive surgery, the disease may be managed without a substantial effect on quality of life [15]. Arguments put forward in favour of offering PND and PGD are that preventive surgery may have a large impact on psychosocial well-being [16] and that families with hereditary cancer syndromes are burdened by their increased risk and deserve the same choices as families with other high-risk hereditary diseases [12, 17].

PND with the intention to terminate the pregnancy of an affected child is likely to reflect the perceived burden of the disease, i.e. future parents will only consider PND when they perceive the disease to be severe and wish to prevent their child from suffering [14]. In the Netherlands, PND for most hereditary cancers is offered as a reproductive option after extensive and careful consultation of the future parents with the clinical geneticist, a psychosocial worker and the gynaecologist [18, 19]. In several countries, PND for cancer syndromes has been performed, as listed in a review from 2006 [1]. However, most of the studies from this review are case descriptions, and papers on consecutive series on the uptake of PND for cancer are sparse.

We previously reported on reproductive decisions of couples at risk of having a child with retinoblastoma (Rb), a rare type of eye cancer in early childhood [3, 4]. Several couples who participated in these studies reported that they had chosen PND to prevent the birth of an affected child with Rb. The present study was conducted to determine how many families have used PND as a reproductive option for Rb since DNA diagnosis for Rb became available in the Netherlands in 1990. To put these data into perspective, we compared the use of PND for Rb to the uptake of PND for four other cancer syndromes with autosomal dominant inheritance.

## Methods

### Design

Comprehensive retrospective study in all (nine) academic diagnostic DNA laboratories within the Netherlands.

### Choice of hereditary cancer syndromes

A comparison was made between Rb and four autosomal dominantly inherited cancer syndromes, i.e. Von Hippel–Lindau disease (VHL), Li–Fraumeni syndrome (LFS), familial adenomatous polyposis (FAP) and *BRCA1*- and *BRCA2*-related hereditary breast ovarian cancer (HBOC). These syndromes were selected as examples of cancer syndromes that may have cancer onset in early childhood (VHL, LFS), adolescence (FAP) or in adult life only (HBOC). Cancer syndromes with <40 families registered at the nine diagnostic DNA laboratories within the Netherlands were not considered due to presumed lack of power in the comparisons. Information on the different cancer syndromes is provided in Box 1.

**Box 1:** Main characteristics of the hereditary cancer syndromes

Retinoblastoma (Rb) is a pediatric malignant tumor of the embryonic neural retina cells, usually diagnosed in the first few years of life [20]. In 40% of cases Rb is heritable caused by a germline *RB1*-mutation. Heritable Rb is an autosomal dominant disease with high penetrance: more than 95% of germline mutation carriers develop Rb. They also have an increased risk of developing other malignancies later in life. Healthy parents with a child with a de novo *RB1* mutation have a 2–3% recurrence risk for their next child, based on possible germline mosaicism.	
Von Hippel–Lindau’s disease (VHL) is caused by mutations in the *VHL*-gene [21]. Its main characteristics are haemangioblastomas of the brain, retina and spinal cord, renal cysts and renal carcinoma, and phaeochromocytoma. Penetrance is high. Expression varies greatly both within and between families. Screening starts usually at the age of 5 years in the Netherlands.	
Li–Fraumeni syndrome (LFS) is associated with germline mutations in the *TP53*-gene and is characterized by an increased risk for a variety of malignancies at young age, sometimes during childhood, including sarcomas, early onset breast cancer, adrenocortical carcinoma, leukemia and brain tumors [22]. No effective preventive measure currently exists for *TP53*-mutation carriers, other than awareness and prompt visit to a physician with unexplained complaints. Recently, annual whole body MRI screening of carriers has started both in the Netherlands and other countries (e.g. see http://clinicaltrials.gov/ct2/show/NCT01464086), although the effect on survival is currently not known.	
Familial adenomatous polyposis (FAP) is caused by mutations in the *APC*-gene, with virtually complete penetrance: close to 100% of carriers develop FAP [23]. Carriers develop extensive polyposis of the colon, leading to colon cancer if untreated. Other features include an increased risk for duodenal polyps and desmoid tumors. Screening for polyps starts from age 10–12 years and preventive colectomy is usually performed in early adulthood.	
Hereditary breast and ovarian cancer (HBOC) is caused by mutations in the *BRCA1*- or *BRCA2*-gene. The lifetime risk for women of developing breast cancer is 40–80% and the cumulative risk of developing ovarian cancer is 11–40% [24]. Breast screening starts at the age of 25 years. Bilateral salpingo-oophorectomy and bilateral mastectomy are offered as preventive measures.	

### Genetic counselling and DNA testing

In the Netherlands, costs for genetic counselling and DNA testing are covered within the national health service, and exclusively carried out by the DNA laboratories of the departments of clinical genetics of the university hospitals. All patients or their parents had at least one informative counselling session at a family cancer clinic with a genetic counsellor before DNA testing was performed. According to standard procedures, oral and written information about the cancer syndrome and the test results were provided. Reproductive options will have been discussed when the at-risk counselee was in the reproductive age.

DNA testing of the different cancer syndromes are apportioned among the nine laboratories; while some genes are diagnostically tested in nearly all laboratories (e.g. *BRCA1/2*), others are tested in only one (e.g. *RB1*).When a pathogenic mutation is detected within a family, DNA testing of subsequent family members will be performed in the laboratory in which the initial genetic diagnosis was made. Therefore the likelihood that family members are tested in two different laboratories is small. All laboratories use databases to keep track of family relationships and test results, including prenatal diagnostic tests.

For this study, a family is defined as all related mutation-positive family members from one family. The age and gender of the mutation carriers have not been registered.

The study has been approved by the Medical Ethics Review Committee of VU University Medical Center (VUMC) Amsterdam and was conducted in accordance with the principles of the Helsinki declaration.

### Questionnaire

For this retrospective study, molecular geneticists of the nine DNA laboratories in the Netherlands were asked the same questions for all five cancer syndromes in May 2013 (end of inclusion):

1. When did DNA diagnostic testing start in your laboratory?
2. How many mutation-positive families are known in your laboratory?
3. How many families have opted for PND since DNA testing became available?
4. What was the date of each PND?
5. How many times has PND been performed per family?

The laboratory was re-contacted when necessary.

### National Retinoblastoma Treatment Center

Since 1991, all newly diagnosed Dutch Rb patients are being treated in the National Retinoblastoma Treatment Center at the VUMC in Amsterdam. This means that the majority of patients and their parents visited the clinical genetics department of VUMC for counselling for Rb. For this study, we registered which clinical genetics department in the Netherlands had requested PND for Rb. For the other hereditary cancer syndromes, there is no central treatment centre, so (pre-PND) genetic counselling was performed in all nine clinical genetics departments.

### Statistical analysis

To test whether PND was performed more or less often in Rb families than in families with each of the other hereditary cancer syndromes, two-sided Fisher’s exact tests were used. A Bonferroni multiple testing correction was applied for the number of tests that were performed (uptake for Rb was compared with four hereditary cancer syndromes); *p* values <0.05/4 = 0.0125 were considered significant.

## Results

Table 1 shows the five hereditary cancer syndromes, the number of mutation-positive families identified in the Netherlands, the total number of PNDs performed per cancer syndrome, and the number of couples that performed PND. PND was performed 35 times for Rb by 22 couples from 22 mutation-positive families (11.8% of 187 mutation-positive families). The percentages of families opting for PND for the other cancer syndromes ranged from <0.2% (HBOC) to 6.5% for VHL.

**Table 1:** Cancer syndromes with number of families known in the Netherlands, number of PNDs and comparison with number of PNDs for retinoblastoma

Cancer syndrome	Number of families with a germline mutation	Number of PNDs	Number of couples that performed PND (percentage of total number of mutation-positive families)^a^	Uptake for Rb compared to other cancer syndrome *p* value
Rb	187	35	22 (11.8%)	
VHL	92	7	6 (6.5%)	0.207
LFS	41	5	2 (4.9%)	0.266
FAP	364	11	6 (1.6%)	<*0.0001*
HBOC	>3000	6	6 (<0.2%)	<*0.0001*

*Rb* retinoblastoma, *VHL* Von Hippel–Lindau disease, *LFS* Li–Fraumeni syndrome, *FAP* familial adenomatous polyposis, *HBOC* hereditary breast and ovarian cancer, *PND* prenatal diagnosis

^a^Of all couples opting for PND, fifteen performed PND more than once. In 41 out of 42 mutation positive families PND was performed by one couple per family. In one family with a p53 mutation two different couples performed PND, here taken as one case

Significant *p* values are in italics

A significant difference in number of PNDs was seen between Rb and HBOC, and Rb and FAP. No differences were seen between PNDs for Rb and VHL and LFS.

In 14 out of 22 couples that used PND for Rb, one of the parents was a carrier of an *RB1* mutation and the risk of a child with Rb was 50%. The other eight families concerned healthy parents with a child with a *de novo RB1* mutation. The recurrence risk for these couples was 2–3%, based on possible germline mosaicism. For the other four cancer syndromes, the risk of an affected child was 50% for all cases. When confining the comparison analysis to Rb cases with a 50% risk, a significant difference in use of PND was still seen between Rb and FAP (*p* = 0.0011) and Rb and HBOC (*p* < 0.0001).

### Number of PNDs per year

**Fig. 1:**
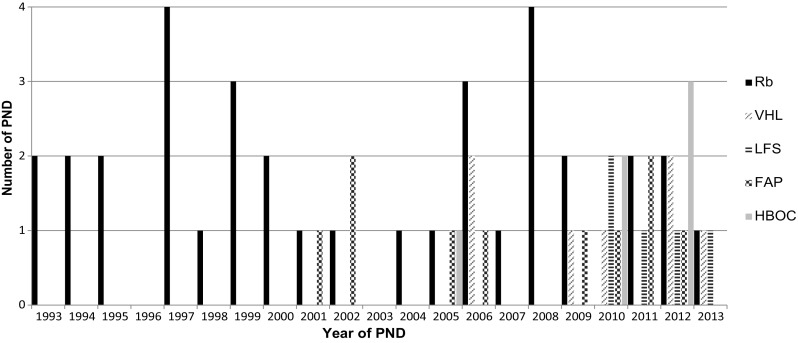
Number of PNDs per hereditary cancer syndrome per year. *Rb* retinoblastoma, *VHL* Von Hippel–Lindau disease, *LFS* Li–Fraumeni syndrome, *FAP* familial adenomatous polyposis, *HBOC* hereditary breast and ovarian cancer, *PND* prenatal diagnosis

In Fig. 1 the number of PNDs for all hereditary cancer syndromes is plotted per year.

The first PND for Rb was done in 1993 and the last PND included in the study was done in 2013. In that period there has been neither a substantial increase nor decrease in the number of PNDs for Rb per year: the number of PNDs per year ranged from zero to four, with a mean of 1.5 per year. The number of PNDs per year for the other cancer syndromes varied between zero and three per syndrome per year. There was a trend towards more PNDs after 2009: eight out of a total of 28 PNDs for these cancer syndromes were performed between 2001 and 2009. The other 20 PNDs were performed between 2009 and 2013.

In 22 out of 35 PNDs for Rb, the pre-PND counselling and the invasive procedure was done in VUMC, where the National Retinoblastoma Treatment Center is located and the remaining thirteen pre-PND counselling and invasive procedures were conducted elsewhere. Pre-PND counselling and invasive procedures for the other cancer syndromes were performed in all participating hospitals, except for one: in the National Cancer Institute no invasive PND procedure is available and those patients were referred to one of the other eight centres.

### Time between start of DNA diagnostic testing and first PND

In Table 2 the year of gene identification, start of DNA diagnostic testing per cancer syndrome is depicted and number of years between the start of DNA diagnostic testing and the year of the first PND. For Rb the first PND was done 3 years after DNA diagnostic testing had been introduced in the Netherlands. For the other cancer syndromes the first PND was performed between 10 and 15 years after DNA diagnosis became available.

**Table 2:** Genes related to the five hereditary cancer syndromes with year of gene identification, year of start of DNA diagnostic testing in the Netherlands, year the first prenatal diagnosis (PND) was performed, and the number of years between the start of testing and the first PND

Cancer syndrome	Related gene	Year of gene identification	Start DNA testing in the Netherlands	First PND	Number of years between start DNA testing and first PND
Rb	RB1	1986 [25]	1990	1993	3
VHL	VHL	1993 [26]	1994	2006	12
LFS	TP53	1990 [27]	1995	2010	15
FAP	APC	1991 [28]	1991	2001	10
HBOC	BRCA1/BRCA2	1994 [29]/1995 [30]	1995	2005	10

*Rb* retinoblastoma, *VHL* Von Hippel–Lindau disease, *LFS* Li–Fraumeni syndrome, *FAP* familial adenomatous polyposis, *HBOC* hereditary breast and ovarian cancer, *PND* prenatal diagnosis

Analysis comparing uptake for Rb to FAP and HBOC was done again, while taking into account the year DNA diagnostic testing became available. Since DNA testing for FAP has been possible since 1991 and the first PND for Rb was performed in 1993, the analysis was unchanged for FAP. For HBOC, uptake of PND for Rb remained significantly different both for all PNDs (*p* value <2.2e−16) and when just comparing PNDs of couples with a 50% risk of a child with Rb (*p* value <1.296e−12).

## Discussion

In this study, relatively large differences in the use of PND between cancer-predisposing syndromes were found. A significantly higher uptake for Rb than for the adult-onset cancer syndromes HBOC and FAP was seen. Uptake of PND did not differ significantly between Rb and two other early onset cancer syndromes VHL and LFS. PND for Rb started just 3 years after DNA diagnostic testing was introduced and uptake has been relatively stable over the years. PND for VHL, LFS, FAP and HBOC started 10–15 years after DNA testing was offered. A trend towards more PNDs for these syndromes after 2009 was noted.

The differences in uptake for PND observed in this study may be explained by several interdependent factors: differences in age of onset of cancer, disease penetrance, risk-reducing options and perceived disease burden, as noted by several authors in papers on PND or PGD for hereditary cancer [1, 5].

When age of onset is in adulthood, prospective parents may have the hope for better treatment options in the future for a carrier child, whereas in the case of childhood-onset the parents’ concerns will be more immediate. For adult-onset cancers, cancer diagnosis of an individual may not have been made until after family planning was completed. Rb is a high-penetrance disease of early childhood, and therefore parents of an affected child with Rb may still be in the reproductive age at the time of diagnosis of an affected child and may opt for PND in a subsequent pregnancy. Furthermore, physicians caring for patients with childhood cancer often have more intense contact with the family and will have more awareness of a possible impact of childhood cancer on family planning and be more knowledgeable about reproductive options than physicians caring for adult-onset cancer patients [31]. Differences in uptake may also be explained by the lack of risk-reducing options for the hereditary cancer syndromes with childhood-onset, apart from screening to detect cancer at an early stage [32]. For HBOC, mastectomy and prophylactic salpingo-oophorectomy can reduce cancer risk substantially, and the same applies to colectomy for FAP.

Lastly, previous studies on reproduction and hereditary cancer considered the perceived disease burden by individuals opting for assisted reproduction a factor of influence on uptake of PND [5]. For Rb, an extra disease burden follows from the intensive ophthalmological screening, which is done under anaesthesia in the first few years of life. Also, patients with heritable retinoblastoma have an increased risk for both a pineoblastoma (also referred to as trilateral retinoblastoma) and second tumours later in life and many have considerable late effects of treatment, e.g. an impaired vision or hearing loss. Perceived disease burden is not always related to a personal history of cancer, but can be shaped by many aspects, like caring for a family member with cancer [1, 5]. In our study of reproductive behaviour of individuals at risk of a child with Rb, the most important factor of influence on reproductive behaviour was perceived risk, not objective risk [4]. Both perceived disease burden and perceived risk may therefore be part of the reason eight parents with a child with a *de novo RB1* mutation opted for PND, in spite of a recurrence risk of <3%.

One of the possible reasons PND uptake for Rb may differ from uptake for FAP and HBOC could be that diagnostic DNA testing of Rb started earlier than for FAP and HBOC. Therefore analysis comparing uptake for Rb to FAP and HBOC was done again, while taking into account the year DNA diagnostic testing became available. This analysis, however, did still show a significant difference between uptake for Rb and uptake for FAP and HBOC.

Observed higher uptake for Rb may be a reflection of differences in counselling between our clinical genetics department (with the highest number of counselees for Rb) and the other eight clinical genetics departments in the Netherlands. However, 13 PNDs and pre-PND counselling for Rb were performed elsewhere. Since there is a close collaboration between the nine clinical genetics departments in the Netherlands, policy towards counselling and PND is much the same.

PND for hereditary cancer has been reported in other countries, although in limited numbers, making it difficult to compare these data to our findings [1]. One paper on the clinical perspective on ethical arguments around PND and PGD for later-onset cancer syndromes from the Regional Genetics Service in Manchester mentioned one couple out of 110 families with FAP known in their centre that had undergone PND, and none from 356 HBOC families [14]. In Canada, PND for Rb is done to enhance early management of *RB1* carrier infants and not with the option to terminate the pregnancy [33]. In the case of an affected child, premature delivery at 36 weeks’ gestation is recommended to be able to treat as early as possible.

One of the limitations of this study is that the number of mutation carriers in the reproductive age in each family was not known. However, since a relatively large number of families were included, we believe that our data on the uptake of PND in the cancer syndromes are by and large reliable. A trend towards increasing uptake of PND for hereditary cancers other than Rb after 2009 was observed. In 2008, PGD for hereditary cancer was temporarily restricted by the Dutch Minister of Health because of an ethical debate on PGD for diseases with a penetrance of <100%, such as HBOC [34]. Public debate in the media about hereditary cancer and reproduction during those years may have alerted at-risk couples to the option of both PGD and PND. Future research will have to determine whether the observed increasing trend of PND uptake over the past 5 years for VHL, LFS, FAP and HBOC will continue.

In conclusion, PND for Rb started many years before it was used for the other hereditary cancer syndromes. PND has been done significantly more often for Rb than for FAP and HBOC. Uptake of PND was not significantly different between Rb and VHL, and Rb and LFS. Early onset, high penetrance, lack of preventive surgery and perceived burden of disease may explain these differences. Knowledge regarding the underlying motives of couples that have opted for PND as a reproductive option is useful to improve care for families with a genetic predisposition for cancer.

## References

[1] Offit K, Kohut K, Clagett B (2006). Cancer genetic testing and assisted reproduction. J Clin Oncol.

[2] Lammens C, Bleiker E, Aaronson N (2009). Attitude towards pre-implantation genetic diagnosis for hereditary cancer. Fam Cancer.

[3] Dommering CJ, van den Heuvel MR, Moll AC (2010). Reproductive decision-making: a qualitative study among couples at increased risk of having a child with retinoblastoma. Clin Genet.

[4] Dommering CJ, Garvelink MM, Moll AC (2012). Reproductive behavior of individuals with increased risk of having a child with retinoblastoma. Clin Genet.

[5] Rich TA, Liu M, Etzel CJ (2014). Comparison of attitudes regarding preimplantation genetic diagnosis among patients with hereditary cancer syndromes. Fam Cancer.

[6] Mitchell C, Nicolaides K, Kingston J (1988). Prenatal exclusion of hereditary retinoblastoma. Lancet.

[7] Ao A, Wells D, Handyside AH (1998). Preimplantation genetic diagnosis of inherited cancer: familial adenomatous polyposis coli. J Assist Reprod Genet.

[8] Harper JC, Geraedts J, Borry P (2013). Current issues in medically assisted reproduction and genetics in Europe: research, clinical practice, ethics, legal issues and policy. European Society of Human Genetics and European Society of Human Reproduction and Embryology. Eur J Hum Genet.

[9] Council of Europe. Background document on preimplantation and prenatal genetic testing. http://www.coe.int/t/dg3/healthbioethic/Activities/07_Human_genetics_en/default_en.asp. Updated 7-5-2015. Accessed 28-3-2016

[10] Quinn GP, Vadaparampil ST, Bower B (2009). Decisions and ethical issues among BRCA carriers and the use of preimplantation genetic diagnosis. Minerva Med.

[11] Wang CW, Hui EC (2009). Ethical, legal and social implications of prenatal and preimplantation genetic testing for cancer susceptibility. Reprod Biomed Online.

[12] Niermeijer MF, de Wert G, Dondorp W (2006). Preimplantation genetic diagnosis for cancer. Lancet Oncol.

[13] Niermeijer MF, de Die-Smulders CE, Page-Christiaens GC, de Wert GM (2008). Genetic cancer syndromes and reproductive choice: dialogue between parents and politicians on preimplantation genetic diagnosis. Ned Tijdschr Geneeskd.

[14] Clancy T (2010). A clinical perspective on ethical arguments around prenatal diagnosis and preimplantation genetic diagnosis for later onset inherited cancer predispositions. Fam Cancer.

[15] Leading edge (2006). Ethics of preimplantation genetic diagnosis for cancer. Lancet Oncol.

[16] van Oostrom I, Meijers-Heijboer H, Lodder LN (2003). Long-term psychological impact of carrying a BRCA1/2 mutation and prophylactic surgery: a 5-year follow-up study. J Clin Oncol.

[17] Ethics Committee of American Society for Reproductive Medicine (2013). Use of preimplantation genetic diagnosis for serious adult onset conditions: a committee opinion. Fertil Steril.

[18] Cobben JM, Brocker-Vriends AH, Leschot NJ (2002). Prenatal diagnosis for hereditary predisposition to mammary and ovarian carcinoma—defining a position. Ned Tijdschr Geneeskd.

[19] Lodder LN, Frets PG, Trijsburg RW (2000). Attitudes towards termination of pregnancy in subjects who underwent presymptomatic testing for the BRCA1/BRCA2 gene mutation in The Netherlands. J Med Genet.

[20] Lohmann DR, Gallie BL (2015) Retinoblastoma. http://www.ncbi.nlm.nih.gov/books/NBK1452/. Updated 19-11-2015. Accessed 22-4-2016

[21] Frantzen C, Links TP, Giles RH (2015) Von Hippel–Lindau Disease. http://www.ncbi.nlm.nih.gov/books/NBK1463/. Updated 6-8-2015. Accessed 14-3-2016

[22] Schneider K, Zelley K, Nichols KE, Garber J (2013) Li–Fraumeni syndrome. http://www.ncbi.nlm.nih.gov/books/NBK1311/. Updated 11-4-2013. Accessed 17-12-2015

[23] Jasperson KW, Burt RW (2014) APC-associated polyposis conditions. http://www.ncbi.nlm.nih.gov/books/NBK1345/. Updated 27-3-2014. Accessed 17-4-2016

[24] Petrucelli N, Daly MB, Feldman GL (2013) BRCA1 and BRCA2 hereditary breast and ovarian cancer. http://www.ncbi.nlm.nih.gov/books/NBK1247/. Updated 26-9-2013. Accessed 17-11-2015

[25] Friend SH, Bernards R, Rogelj S (1986). A human DNA segment with properties of the gene that predisposes to retinoblastoma and osteosarcoma. Nature.

[26] Latif F, Tory K, Gnarra J (1993). Identification of the von Hippel–Lindau disease tumor suppressor gene. Science.

[27] Malkin D, Li FP, Strong LC (1990). Germ line p53 mutations in a familial syndrome of breast cancer, sarcomas, and other neoplasms. Science.

[28] Kinzler KW, Nilbert MC, Su LK (1991). Identification of FAP locus genes from chromosome 5q21. Science.

[29] Miki Y, Swensen J, Shattuck-Eidens D (1994). A strong candidate for the breast and ovarian cancer susceptibility gene BRCA1. Science.

[30] Wooster R, Bignell G, Lancaster J (1995). Identification of the breast cancer susceptibility gene BRCA2. Nature.

[31] Brandt AC, Tschirgi ML, Ready KJ (2010). Knowledge, attitudes, and clinical experience of physicians regarding preimplantation genetic diagnosis for hereditary cancer predisposition syndromes. Fam Cancer.

[32] Julian-Reynier C, Chabal F, Frebourg T (2009). Professionals assess the acceptability of preimplantation genetic diagnosis and prenatal diagnosis for managing inherited predisposition to cancer. J Clin Oncol.

[33] Gallie B (2009). Canadian guidelines for retinoblastoma care. Can J Ophthalmol.

[34] Sheldon T (2008). Netherlands debates screening for breast cancer. BMJ.

